# Nanomedicine for renal cell carcinoma: imaging, treatment and beyond

**DOI:** 10.1186/s12951-022-01761-7

**Published:** 2023-01-03

**Authors:** Ruolin Wu, Keshan Wang, Yongkang Gai, Mengting Li, Jingjing Wang, Chenyang Wang, Yajing Zhang, Zhiwei Xiao, Dawei Jiang, Zairong Gao, Xiaotian Xia

**Affiliations:** 1 Department of Nuclear Medicine, Union Hospital, Tongji Medical College, Huazhong University of Science and Technology, No. 1277 Jiefang Avenue, Wuhan, 430022 Hubei People’s Republic of China; 2 Hubei Province Key Laboratory of Molecular Imaging, Wuhan, China; 3 Key Laboratory of Biological Targeted Therapy, The Ministry of Education, Wuhan, China; 4 Department of Urology, Union Hospital, Tongji Medical College, Huazhong University of Science and Technology, Wuhan, People’s Republic of China; 5 Department of Nuclear Medicine, Zhongnan Hospital of Wuhan University, Wuhan, China

**Keywords:** Renal cell carcinoma, Nanomedicine, Diagnosis, Treatment, Theranostics

## Abstract

The kidney is a vital organ responsible for maintaining homeostasis in the human body. However, renal cell carcinoma (RCC) is a common malignancy of the urinary system and represents a serious threat to human health. Although the overall survival of RCC has improved substantially with the development of cancer diagnosis and management, there are various reasons for treatment failure. Firstly, without any readily available biomarkers, timely diagnosis has been greatly hampered. Secondly, the imaging appearance also varies greatly, and its early detection often remains difficult. Thirdly, chemotherapy has been validated as unavailable for treating renal cancer in the clinic due to its intrinsic drug resistance. Concomitant with the progress of nanotechnological methods in pharmaceuticals, the management of kidney cancer has undergone a transformation in the recent decade. Nanotechnology has shown many advantages over widely used traditional methods, leading to broad biomedical applications ranging from drug delivery, prevention, diagnosis to treatment. This review focuses on nanotechnologies in RCC management and further discusses their biomedical translation with the aim of identifying the most promising nanomedicines for clinical needs. As our understanding of nanotechnologies continues to grow, more opportunities to improve the management of renal cancer are expected to emerge.

## Background

Of the kidney's numerous significant functions, electrolyte homeostasis, elimination of waste metabolites from the blood, and regulation of blood pressure via the renin–angiotensin–aldosterone axis are crucial for survival [1]. Unfortunately, kidney cancers are a serious threat to the health and life of human beings. The latest cancer statistics of the American Cancer Society show that kidney and renal pelvis cancers are among the top ten diagnosed cancers [2], leading to an estimated 13,920 deaths in 2022 [3]. Among all urological malignancies, incidence of renal cell carcinoma (RCC) ranked the third [4], accounting for 3% of adult malignancies and 80–90% of renal tumors, of which the male-to-female patient ratio is approximately 2:1 [5]. However, compared to other cancers, the mechanism underlying the pathogenesis of kidney cancer is poorly understood. Computed tomography (CT), magnetic resonance imaging (MRI), and ultrasonography (US) are traditionally used for detection of non-invasive malignancies. Because of highly heterogeneous in kidney cancer imaging, these approaches have limited sensitivity and ability to provide specific and functional information of RCC at an early stage [6]. Besides kidney cancer treatment has historically consisted on three main approaches: surgery, chemotherapy, and radiation therapy [7]. But the most dreaded consequences include a lack of effectiveness in chemotherapy and radiation therapy [8], as well as a high recurrence rate of up to 40% after surgical resection [9]. During the past decades, various novel therapeutic modalities, including immunotherapy and targeted therapy, have been discovered and implemented clinically to provide patients with improved therapeutic outcomes. Nevertheless, there are still some limitations of these therapies, including the complex nature of the molecular targets, severe side effects and high prices [10]. The delivery of medicine and their responses in tumor areas remains challenging due to the complexity and heterogeneity of the tumor microenvironment.

With the advent of the nanotechnology era, strategic application of nanotechnologies to pharmaceutical research has led to successful development of nanomedicine. Therefore, it is essential to develop different options with nanotechnology that enable the early detection of kidney malignancies, along with accurate diagnoses and precise treatment strategies. Nanomaterials possess potential advantages in diagnosis and therapy of tumors, allowing for the simultaneous scanning of multiple biomarkers in liquid biopsies and cell cultures, selective drug delivery to cancerous cells, avoidance of potential toxicity to normal cells, and improved biological profiles to maximize bioavailability in vivo [6, 11, 12]. Even so, there was not given enough attention for RCC during the initial exploration phase of nanomedicine study. This was the reason that many potentially nanomedicine exhibited poor pharmacokinetics especially with respect to the kidneys after being introduced into a living subject. Many nanomedicines also present challenges with instability, potential toxicity, cytotoxicity, immune response, and chronic inflammation [13]. These are so dangerous for patients who have been shown to be accompanied by renal dysfunction when they were diagnosed with RCC.

Fortunately, several studies have discovered that some nanoparticles have an inherent propensity for glomerular deposition, which makes it possible to deliver drugs to the kidney [14–16]. Nanomedicine has made a lot of technological advances. For RCC management, it might be used to improve its effectiveness in the following ways: First, diagnostic assays based on nanoscale sensors enable biomarker detection at the femtomolar-level to compose diagnostic profile for renal cancer patients [17]. Second, well-designed nanoparticles can deliver formulations or drugs across traditional biological barriers in the body and be directed to specific cell types within target organs via active/passive targeting. This allows for molecular imaging, diagnosis and treatment of kidney cancer [18]. Third, nanoparticles have the potential to bypass chemo-drug or radiotherapy resistance mechanisms and lower cancer-therapy-related adverse effects [19]. Moreover, nanoparticles can carry two or more therapeutic agents to achieve synergistic results and can be tuned to provide suitable circulation times [20]. The implications of this work may break this bottleneck of nanomedicines for renal disease management.

**Fig. 1:**
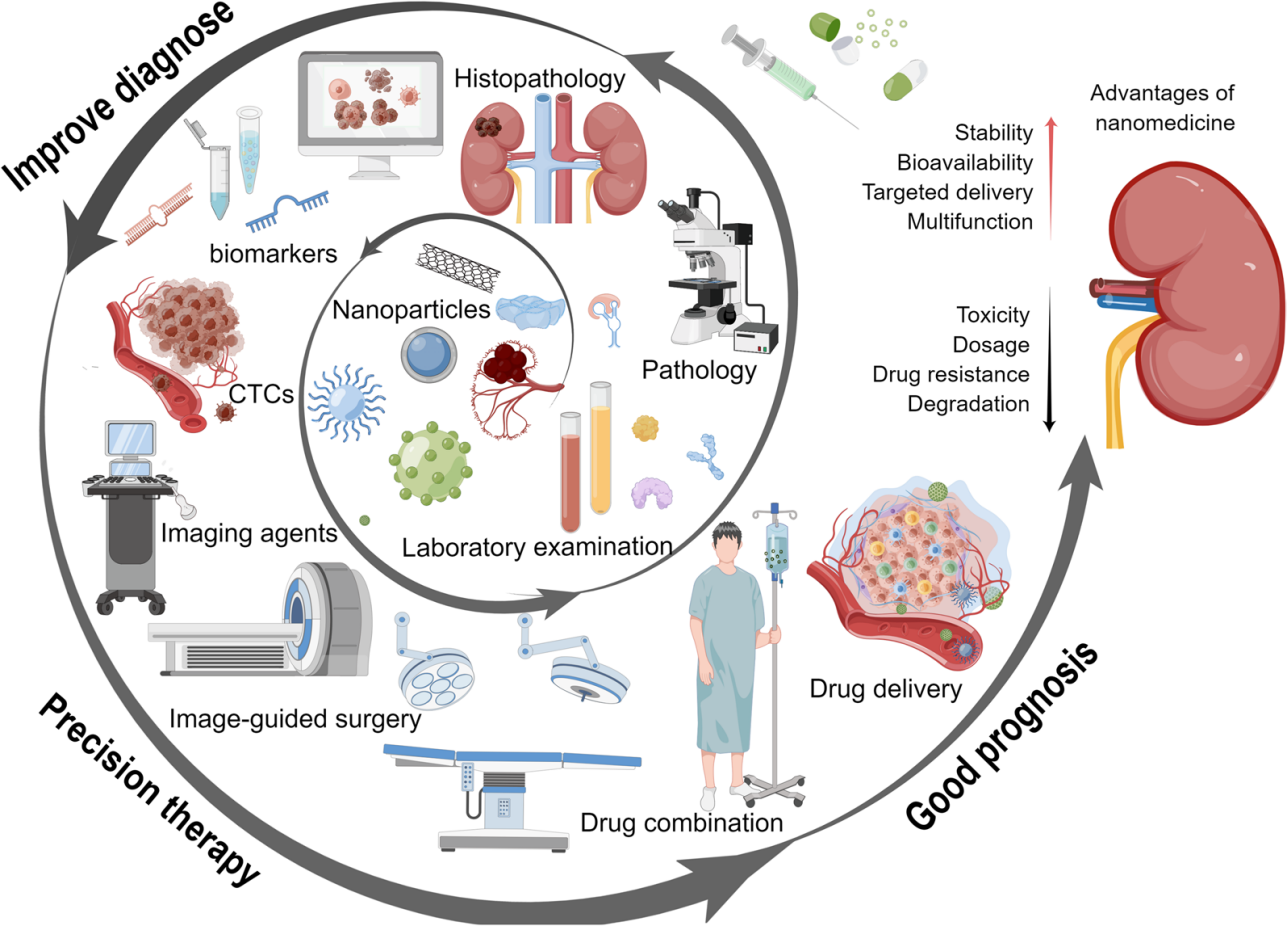
Scheme illustrating the latest advances of nanomedicines in diagnostic and therapeutic interventions of RCC

Herein this article, we systematically scrutinize the latest advances in nanotechnologies in diagnostic and therapeutic interventions of RCC, with special focus given to their applicability and potential clinical translation (Fig. 1). Furthermore, we demonstrate how to overcome the inherent and contextual limitations of traditional clinical measures.

## Diagnosis of renal cell carcinoma using nanomedicine

In clinical practice, RCC often develops with no signs or symptoms. In most cases, it is diagnosed as an incidental finding and is referred to as the “silent disease” [21]. The typical symptoms of RCC patients, such as pain, lumps, and hematuria, account for only 10% of cases, and their manifestation suggests either aggressive or advanced disease stages [22]. A diagnosis based on history and physical examination alone is difficult, even in high-risk patients. The on-time diagnosis has been severely impeded since there are currently no biomarkers available for clinical diagnosis, early detection and follow-up of the disease. Luckily, Colaianni et al. applied gold nanowires with good results for small peptide analysis [23]. Nanoflowers of Au@MnO were used for analysis of small and large molecules of cancer cell lysates [24]. A MoS_2_ quantum dot (QD) was prepared for long-term tracking of living cells [25]. Nowadays, there are accumulating studies on the coalescing of nanotechnology and conventional approach to improve diagnostic efficiency. In this section, we provide a summary and discussion. Diagnosing and monitoring kidney cancer is dependent on appropriate laboratory examination of bodily fluids, pathologic examinations, and imaging assessments, which can help to obtain more information concerning kidney cancer metabolism (Table 1).

**Table 1:** The existing nanomedicine for diagnosis of renal cell carcinoma

Diagnostic type	Diagnostic agent	Disease model	Advantages	References
Laboratory examination of bodily fluids	Gold nanoparticles-assisted laser desorption/ionization mass spectrometry	Human study	Distinguish types, grades and stages of human RCC	[37, 38]
	High-resolution proton nuclear magnetic resonance spectroscopy and silver-109 nanoparticle enhanced steel target laser desorption/ionization mass spectrometry	Human study	Have potential for use in clinical prognosis and/or diagnosis	[26, 39]
Pathology	Gold nanoparticle enhanced target	Human study	Differentiate between normal and cancerous renal tissue	[40]
	Silver nanoparticle enhanced target	Human study	Distinguish healthy and cancer tissue	[41–43]
	Magnetic nanoparticle with immobilized trypsin	Human study	Differentially cluster renal oncocytoma and chromophobe RCC	[44]
	Peptide-coated Au clusters with intrinsic red fluorescence and a specific mass signal	In vitro, in vivo and human study	Assess the risk of primary tumor invasion/metastasis	[47]
	A new nanopore-based detection scheme	In vitro	The detection of miRNA-204 and miRNA-210 related to the RCC	[17]
	Biotin-streptavidin binding and fluorescence active magnetic nanocarriers	Human study	Detectable low levels of miRNA15a through miRNA capturing nanocarriers	[54]
	EVs detected by nanoparticle tracking analysis	In vitro and human study	CA9, CD70, and CD147 could represent promising markers to identify tumor-specific EVs in RCC	[55]
	Shell-isolated nanoparticle-enhanced Raman spectroscopy in microfluidic device	In vitro	Improve the detection accuracy and sensitivity of analyzed circulating tumor cells	[57]
	Integration of dendrimer-mediated multivalent binding, a mixture of antibodies, and biomimetic cell rolling	In vitro	Improve the capture of RCC-CTCs by up to 80%	[58]
Imaging	Anti-G250 nanobody-functionalized targeted nanobubbles for ultrasound	In vitro and in vivo	Specifically bind to G250-positive RCC cells and enhance ultrasound imaging of xenografts	[64]
	Targeted nanobubbles carrying CAIX polypeptides/aptamer	In vitro and in vivo	Enhance image contrast in CAIX-positive tumor tissues	[68, 69]
	Lymphotropic nanoparticle-enhanced magnetic resonance imaging	Human study	Accurately distinguish benign from malignant lymph node involvement in patients with RCC	[75]
	Liposomes loaded with hydrophilic magnetite nanoparticles	In vitro and in vivo	Used as the potential contrasting agents for MRI	[76]
	mAb G250-SPIO molecular nanoprobe	In vitro	Used in the specific labeling of RCC cells successfully	[77]
	AS1411 Aptamer Modified Mn-MoS_2_ QDs	In vitro and in vivo	Fluorescently label RCC cells and present a specific MRI signal enhancement in the tumor region	[18]
	Hydrophilic manganese oxide nanoparticles modified AS1411 aptamer (AS1411-PEG-MnO nanoprobe)	In vitro and in vivo	High T_1_ MRI relaxivity, significant accumulation and prolonged retention in tumor	[79]
	Fe_3_O_4_@mSiO_2_/PDDA/BSA-Gd_2_O_3_ nano-complex	In vitro and in vivo	Good performance for tumor cell targeting and potential as a T_1_-T_2_ dual-mode CA	[80]
	^99m^Tc-nanocolloid	Human study	Sentinel node mapping of renal tumors	[83–86]

### Laboratory examination of bodily fluids

There is a continuous exchange of metabolites across different types of tissues and bodily fluids. Thus, variations in biofluid metabolomes reflect changes in tissue metabolism. Metabolic profiling of biofluids, usually serum, generates a biochemical fingerprint of small molecule metabolites, allowing for the discovery and characterization of relevant cancer biomarkers [26]. RCC originates in the tubular epithelium and causes in the release of particular metabolites into its lumen. Since genetic studies have demonstrated that RCC is a metabolic disease, an increasing number of research are concentrating on profiling serum, plasma, urine, and tissue samples from kidney cancer patients [27]. These processes can be used to differentiate the metabolome of sick and healthy people [28]. Beside this, body fluids such as serum and urine, can be obtained with minimal discomfort, thus are the most preferable materials for biomarker examination in cancer research. A total of 71 variables were useable as potential markers but the identification of chemical compounds was only successful for only a few of them [29]. Biological fluids are characterized by high variability among individuals based on age, gender, nutrition, and activity level, which have already been proven by researchers who used various types of liquid chromatography combined with mass spectrometry (MS) [30–34]. Furthermore, biomarkers are often present at extremely low concentrations [32].

Over the past decade, a search of literature has shown, gold/silver nanostructures are among the most frequently used for laser MS [24, 35]. These have been proven to be suited for low and medium polar compounds, produce much lower chemical background and allow more precise internal calibration [36]. Sensitive analytical methods have been developed, enabling better understanding of the metabolic changes over the course of kidney cancer phenotypic development. Several research groups have developed in vitro RCC detection nanoplatforms by examining bodily liquids. Adrian Arendowski et al. developed gold nanoparticle enhanced target (AuNPET) surface assisted-laser desorption/ionization (SALDI) MS for in vitro assessment of RCC, and successfully differentiated serum/urine samples from healthy volunteers and patients with advanced RCC [37, 38]. In another study, Joanna Nizioł et al. further improved the detection accuracy and constructed a prediction model construction by comprehensive high resolution proton nuclear magnetic resonance spectroscopy (^1^H NMR) and silver-109 nanoparticle-enhanced steel target laser desorption/ionization mass spectrometry (^109^AgNPET LDI MS) approaches [26, 39]. Increased level of glucose and decreased levels of choline, glycerol, glycine, lactate, leucine, myo-inositol, and 1-methylhistidine in serum are found closely associated with RCC at different stages [26].

Metabolic profile in kidney cancer detected by MS with nanoparticles better highlights a separation trend between the two groups, although subtle, indicating that there exist inherent metabolic profile differences between kidney cancer and healthy controls. These works will provide frameworks to expand biomarker discovery.

### Pathological examination

Metabolite concentration fluctuations indicate alterations in cellular metabolism. Cell cultures and tissues are the best materials for biomarker and metabolomics research. Tissue samples must be collected in an invasive approach (surgery, biopsy).

#### Histopathology

Because of high risk of positive surgical margins and unfavorable prognosis, the European Association of Urology guidelines recommend to favor radical nephrectomy over nephron-sparing surgery/partial nephrectomy for large renal tumors [7]. However, after confirming the nonmalignant nature of the tumor, possibly with intraoperative MS examination, when risk of positive margins is not clinically significant, nephron-sparing surgery could be the technique of choice. Those patients would benefit from lesser decline of renal function after surgery and have better quality of life.

However, well-known drawbacks of matrix-assisted LDI (MALDI) include numerous chemical background peaks in the low-mass region due to the presence and ionization of the applied matrix, the frequent need for external mass calibration, low mass resolution and accuracy due to the thickness of the tissue samples. To circumvent these limitations, Joanna Nizioł et al. demonstrated that AuNPET SALDI MS method for analysis and imaging can clearly differentiate between normal and cancerous renal tissue [40]. This magnitude of difference in octadecanamide ion intensity may be an extremely desirable feature when it comes to cancer tissue diagnosis. The mentioned effect was mostly likely caused by the strong affinity of low-polarity compounds for gold nanoparticles. Silver ions generated by laser irradiation of silver nanoparticles have different ionic radii and electron affinity than gold ions, resulting in a differential cationization efficiency for a given analyte. Furthermore, some researchers suggest that silver nanoparticles have a stronger affinity for low-polarity molecules. Later studies established that LDI MSI on AgNPET enabled rapid visualization of the differences between the RCC and the healthy part of the kidney tissue allowed to distinguish healthy and cancer tissue without the involvement of a pathologist [41]. To avoid artifacts that are present in full scan MS mode, infrared laser ablation-remote-electrospray ionization (LARESI) platform on AgNPET was developed and employed in imaging of target metabolites in human kidney cancer tissue [42]. The acquired MS images revealed significant differences in abundances of selected metabolites between cancerous and noncancerous regions of the kidney tissue. Further study obtained better results that metabolic and elemental profiling of tumor and adjacent normal human kidney tissue from patients with kidney cancer was undertaken using three different analytical platforms (NMR, ICP-OES and LDI MS) [43]. Besides, diagnosis of chromophobe RCC is complicated by morphological and histological features that overlap with renal oncocytoma, a benign neoplasm that occurs at a similar frequency (5%) but has a positive prognosis and does not require aggressive treatment. A novel analytical approach was proposed to discriminate the extract containing the most discriminating features by using ultrasonic assisted in-solution digestion using magnetic nanoparticle with immobilized trypsin, nano-HPLC and high-resolution mass spectrometry (nano-LC-HR-MS) [44]. Protein digestion by trypsin is critical in proteomic analysis. Due to trypsin's poor stability and autolysis, current protein digestion technology in proteomic processing is time-consuming, tedious, and non-automated. Strongly trypsin immobilized magnetic nanoparticles exhibited a high efficiency for the protein digestion [45].

To date, pathologic methods mainly provide information on differentiation/proliferation and potential drug therapy biomarkers of primary tumors. rather than precisely reveal tumor regional invasion and distant metastasis in the body. Antibody-related immunohistochemistry (IHC) and immunofluorescence (IF) are key methods to assess biomarkers of tumor tissue sections in clinical applications [46]. However, these methods have difficulty in quantitatively detecting MT1-MMP as the blurred color changes in IHC and IF. Xiangchun Zhang et al. developed a precise visible quantification method to detect MT1-MMP in primary tumor tissue sections by peptide-coated Au clusters with intrinsic red fluorescence and a specific mass signal [47]. By observing and quantifying the MT1-MMP expression level in human renal carcinoma tissue sections, this study assessed the risk of primary tumor invasion/metastasis. The accuracy of this pathologic method was verified by CT/MRI molecular imaging of cancer patients and traditional pathologic studies of primary tumor tissues.

#### Liquid biopsies

MicroRNAs (miRNAs) are small noncoding RNAs that play a critical role in gene regulation. Recently, traces of cancer-related miRNAs have been identified in body fluids, which make them remarkable noninvasive biomarkers. For its excellent sensitivity and specificity, qRT-PCR technology is currently regarded as the "gold standard" for miRNA detection [48]. However, this technique requires time-consuming and expensive amplification steps, along with labeling and enzymatic reactions. Moreover, the design of the primers for small miRNA sequences has been reported to be challenging [49]. A new PCR-free biosensor-based technology such as the nanopore-based sensing has been developed for miRNA analysis. Nanopores have gained significant attention in the field of genome sequencing, molecular sensing, and medical diagnostics due to their intrinsic ultrasensitive, PCR-independent, truly reagentless, and rapid detection criteria. Yuqian Zhang et al. elucidated a sensitive and robust nanopore-based detection scheme utilizing a borosilicate micropipette and an assay of complementary γ-peptide nucleic acid (γ-PNA) probes conjugated to polystyrene beads to accurately detect miRNA-204 and miRNA-210 fragments related to RCC against the small RNA background [17]. The limit of detection for miRNA-204 and miRNA-210 were demonstrated as 1 and 10 fM, respectively.

Besides, miRNA15a biomarker of RCC is also high potential [50, 51]. But its clinical application is considerably hampered by the insensitive nature of the detection methods and low concentration of biomarker in samples that is aggravated by the high level of contamination due to other solutes present in body fluids. To enhance the capture and reliable detection of miRNA at low concentrations, nanocarrier based approaches offer unprecedented progress in prognosis of various diseases [52]. Nanocarriers are nanomaterials with particle size typically less than 500 nm in diameter and are frequently used as transport vehicles for other substances [53]. Alexander M Renner et al. reported a non-invasive quantitative approach through biotin-streptavidin binding and fluorescence active magnetic nanocarriers that ensured prompt isolation, enrichment and purification of the biomarker miRNA15a from urine [54]. This approach involved the chemically engineered magnetic nanocarriers equipped with surface-attached ss-oligonucleotides, which can be used to separate tumor-related miRNA15a within a few hours. Besides artificial nanosystems for RCC detection, extracellular vesicles (EVs) are secreted by healthy and tumor cells and are involved in cell–cell communication. Tumor-released EVs could represent a new class of biomarkers from liquid biopsies. Transmission electron microscopy (TEM) and nanoparticle tracking analysis (NTA) reflected the size distribution of exosomes. Dirk Himbert et al. found cell line-dependent EV size distributions (as detected by NTA) and the typical protein expression patterns of exosome markers [55]. They demonstrated that the differences regarding the measured sizes of the exosomes in TEM and nanoparticle tracking analysis NTA presumably resulted from the more effective bundling of small exosomes on the coal-copper grid that was used for the TEM. Therefore, NTA is the preferred method for determining the size distribution of exosomes in solution. CA9, CD70, and CD147 could represent promising markers to identify tumor-specific EVs in RCC. Based on these results, further investigations will focus on the development of nanotechnology to enrich tumor-specific EVs from body fluids using these promising markers.

It is well known that isolation and detection of circulating tumor cells (CTCs) from human blood plays an important role in non-invasive screening of cancer evolution and cancer therapy selection [56]. The current methods of CTCs analysis usually utilizing both isolation and detection stages, which are usually completed using separated time-consuming technologies and/or expensive equipment. Niciński et al. presented a new strategy based on surface-enhanced Raman spectroscopy (SERS) to detect CTCs from blood samples in microfluidic chip. The silver nanoparticles coated with an ultrathin shell of silica, namely Ag@SiO_2_, was used to improve the detection accuracy and sensitivity of analyzed tumor cells via shell-isolated nanoparticle-enhanced Raman spectroscopy [57]. The proposed approach challenged the current multi-steps CTCs detection methods in the terms of simplicity, sensitivity, invasiveness, and also prevented the defragmentation/damage of tumor cells and thus lead to improved accuracy. Additionally, Jiyoon Bu et al. further elaborated a novel capture platform to detect RCC-CTCs through integration of dendrimer-mediated multivalent binding, a mixture of antibodies, and biomimetic cell rolling [58]. Capture antibodies were conjugated to dendrimers with a high density of coverage and facilitate multivalent binding at the nanoscale. These results further confirmed that the dendrimer-mediated multivalent binding effect substantially increased adhesion between RCC cells and the capture surface, enhancing the overall capture efficiency of the RCC cells.

Nanoparticles can be utilized not only in histopathology but also in MS to detect low amounts of substances, identify differences, and perform quantitative analysis of a substance's expression in primary tumor tissue. Importantly, nanoparticles have proven to be very effective at capturing traces of miRNA associated with RCC as well as a wide range of CTCs in liquid biopsies.

### Imaging

While in vitro detection of RCC using nanotechnologies advances rapidly, direct visualization of cancerous activities in vivo remains a major challenge for RCC diagnosis. Traditional medical imaging suffers from poor tumor specificity and technical limitations. Nanotechnology has thus been applied for imaging of cancer cells, tumor tissues, and renal mass. Commonly used clinical imaging techniques, including ultrasonography, MRI, and nuclear medicine, can all be facilitated with ever-evolving nanotechnologies in the era of cancer precision medicine, especially RCC. In this section, we scrutinize clinical imaging modalities and discuss potential applications of nanotechnology for RCC imaging.

#### Ultrasonography

Contrast-enhanced ultrasound is helpful because it is easy to use, doesn't use radiation, and can show changes in real time [59]. With the advancement of contrast-enhanced ultrasound, ultrasound molecular imaging (USMI) has arisen [60]. Several studies have shown that targeted ultrasound contrast agents (CAs) with unique antibodies or ligands can bind to specific targets in tissues or disease lesions, allowing molecular or cellular ultrasound imaging [61, 62].

**Fig. 2:**
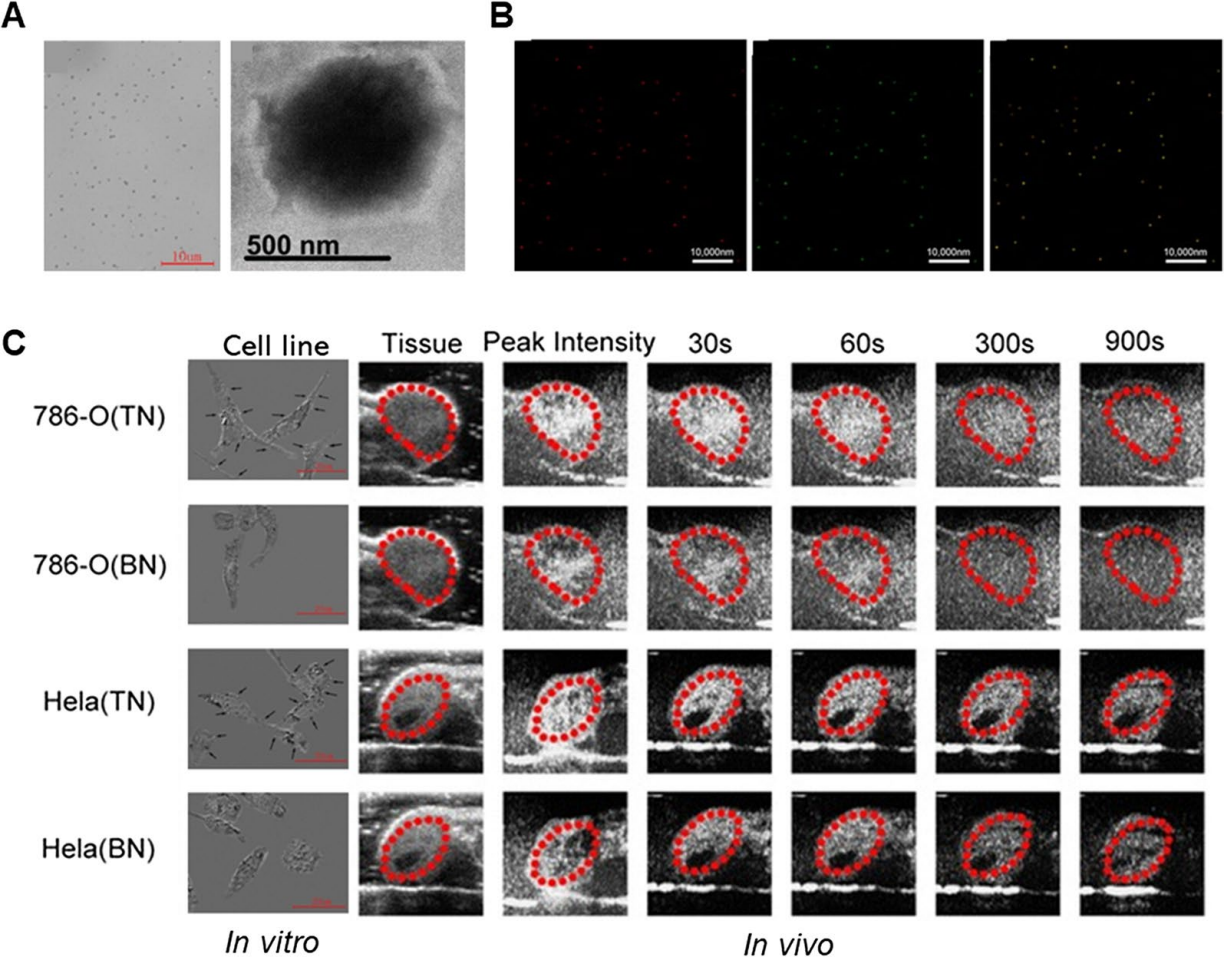
Targeted nanobubbles carrying CAIX polypeptides for targeted binding to a variety of malignant tumors were constructed, and targeted binding ability and ultrasound imaging effect in different types of tumors were evaluated. **A** The image of targeted nanobubbles under an optical microscope and transmission electron microscope. **B** Merged image confirms that biotin-streptavidin system successfully linked the polypeptides to the surfaces of targeted nanobubbles validated by dual-fluorescence. **C** Targeted nanobubbles could gather around CAIX-positive cells (786-O and Hela cells). Peak intensity and duration time of targeted nanobubbles and blank nanobubbles were different in CAIX-positive transplanted tumor tissues in vivo. Figure adapted from Lianhua Zhu et al. [68]

G250 antigen is a transmembrane protein that is highly expressed in most RCCs and is not expressed in healthy kidneys [63]. Zhiping Yu et al. reported that anti-G250 nanobody-functionalized nanobubbles (anti-G250 NTNs) were prepared by coupling anti-G250 nanobodies to lipid nanobubbles to improve the discrimination between benign and malignant renal masses using ultrasound imaging [64]. Importantly, results showed that the antigen–antibody reaction enhanced the stability of the anti-G250 NTNs and their binding to tumor cells, resulting in higher aggregation and retention of the anti-G250 NTNs in the G250-expressing xenografts at levels approximately tenfold those observed in non-G250-expressing xenografts. The anti-G250 NTNs could significantly enhance the ultrasound imaging of G250-expressing xenografts compared with blank nanobubbles. However, most targeted nanobubbles only achieve USMI in blood pool or one type tumor. Linking ligands that have specific targets in common with a wide range of cancers to ultrasound CAs is necessary to make targeted ultrasound CAs that gather in tumor tissues and achieve USMI for a wide range of malignancies. Carbonic anhydrase IX (CAIX) is highly expressed on cell membranes of various malignant solid tumors, making it an ideal target for USMI [65–67]. Lianhua Zhu et al. further investigated that targeted nanobubbles carrying CAIX polypeptides/aptamer can specifically enhance image contrast in CAIX-positive transplanted tumor tissues [68, 69]. The number in CAIX-positive xenograft tumor tissues was significantly different between targeted and non-targeted nanobubbles, and targeted nanobubbles could gather around CAIX-positive tumor cells. Moreover, IF not only confirmed targeted nanobubbles could pass through blood vessels to enter in tumor tissue spaces, but also clarified imaging differences of targeted nanobubbles in different types of transplanted tumor tissues. These will potentially be used in early diagnosis of a variety of solid tumors derived from various organs (Fig. 2).

Currently, microbubbles are currently commonly utilized as an ultrasound CA; however, they cannot target tumor cells or image the tumor selectively because they cannot penetrate blood vessels to reach the tissue space. Furthermore, microbubbles exhibit low stability and have a short half-life [61]. Nanobubbles with small particle size, strong penetration, and long imaging time can aggregate in the extravascular spaces via the enhanced permeability and retention effect [70]. Furthermore, targeting of nanobubbles has been achieved by attaching monoclonal antibodies against cell membrane antigens to the surfaces of the nanobubbles. Targeted nanobubbles can penetrate the tumor vasculature and achieve USMI of tumor parenchymal cells [70, 71].

#### Magnetic resonance imaging

Magnetic resonance imaging (MRI), offering superior resolution, unlimited penetration into tissue and no ionizing radiation, is one of the best strategies used in the clinic to diagnose soft tissue alterations, especially cancer [72]. An important prognostic factor in staging RCC lies on the lymph node (LN) involvement, where the presence of nodal metastases was found strongly associated with decreased life expectancy [73]. Current imaging strategies continue to improve in their capacity to discriminate metastatic LN involvement, but remain low in sensitivity and specificity due to intrinsic restrictions on the size of detectable nodal metastases [74]. Lymphotropic magnetic nanoparticles (MNP), such as ferumoxtran-10 consist of a monocrystalline superparamagnetic iron core and are coated with a dense packing of dextran derivatives. Alexander R Guimaraes et al. assessed lymphotropic nanoparticle-enhanced magnetic resonance imaging (LNMRI) that uses MNP can accurately distinguish benign from malignant LN involvement in patients with RCC [75]. Specific LN uptake occurred via slow extravasation from the vascular space into the interstitial space, lymphatic transport to LNs, macrophage phagocytosis, and intracellular entrapment, which produced the subsequent magnetic properties on MRI. The results were encouraging and warranted a larger, prospective clinical trial although in a relatively small sample size.

**Fig. 3:**
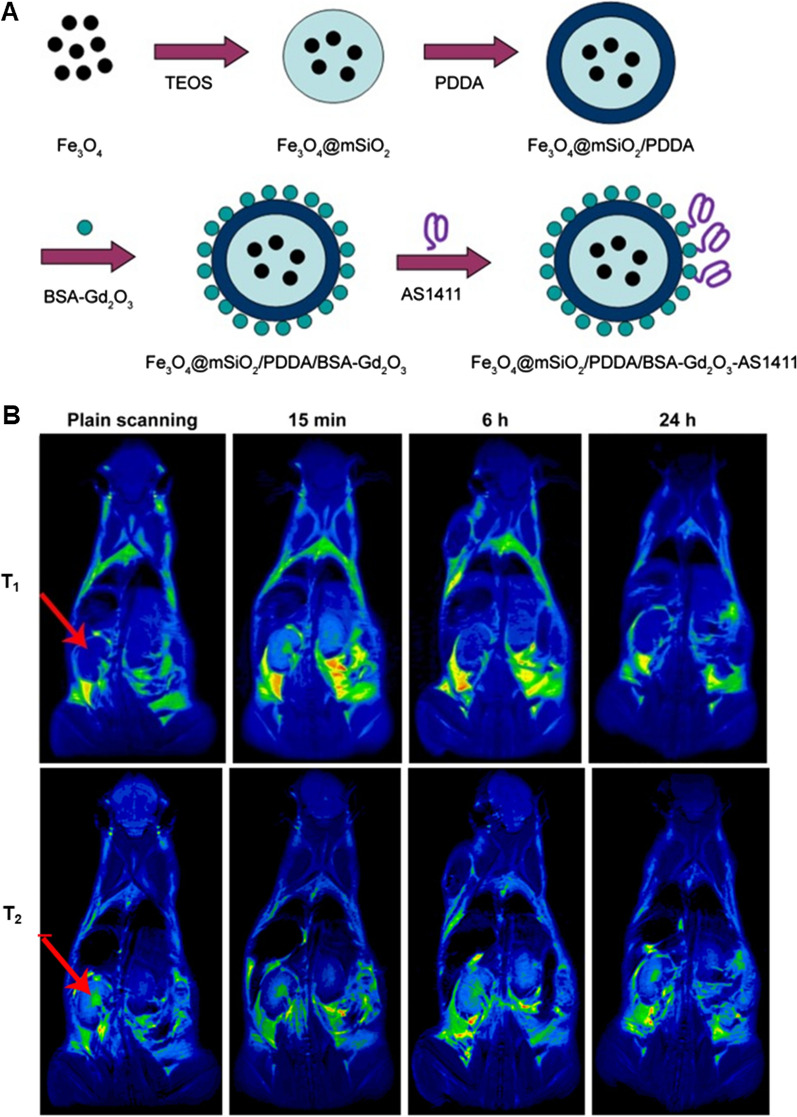
Fe_3_O_4_@mSiO_2_/PDDA/BSA-Gd_2_O_3_ nano-complex was developed as a T_1_-T_2_ dual-mode contrast agent. The combination of T_1_ and T_2_ contrast agents can integrate the high tissue resolution of T_1_ mode contrast imaging and the high feasibility of softer tissue detection of T_2_ mode contrast imaging. **A** Schematic illustration of the fabrication process of the nanoprobes. **B** T_1_-weighted and T_2_-weighted in vivo MRI images of mice post-injection of the nanocomplex at different time points (0, 15 min, 6 h, and 24 h). Figure adapted from Jingjing Li et al. [80]

German et al. fabricated magnetic fluid-loaded liposomes (MFLs) using MNPs and natural phospholipids via the thin film hydration method followed by extrusion [76]. MFLs increased the longitudinal relaxation time (T_1_) and decreased the transverse relaxation time (T_2_) of protons. Compared to pure maghemite nanoparticles, liposomes loaded with MNPs offer a number of benefits, particularly the higher sensitivity to the magnetic field due to the highest magnetic permeability among the iron oxides and the ability to perform photocatalytic reactions. In addition, a sensitive and specific molecular MRI (mMRI) probe plays the most important role in this technique. Cailuan Lu et al. designed that superparamagnetic iron oxide (SPIO) nanoparticles and monoclonal antibody (mAb) G250 were conjugated as mMRI probe [77]. The main advantages of SPIO nanoparticles as MRI CA included their high signal strength, longer lasting contrast enhancement and relatively low cytotoxicity. Additionally, the iron released from degrading SPIO nanoparticles can also be metabolized by body, lowering the risk of long-term cytotoxicity. The fabricated nanoprobe could identify RCC cells sensitively and precisely due to the targeting capacity of mAb G250. The study demonstrated the promise of G250-mAb-SPIO nanoprobe as a molecular magnetic resonance imaging probe for the early diagnostic imaging of RCC that overexpresses the G250 receptor. Also, AS1411 was the first aptamer to be used in clinical trials for the treatment of human cancer [78]. Shaohui Zheng et al. found that the prepared Mn-MoS_2_ QDs exhibited excellent aqueous property, intense fluorescence, low toxicity, high quantum yield of 41.45% and high T_1_ relaxivity of 16.95 mM^−1^ s^−1^. After conjugation with AS1411 aptamer, the AS1411-Mn-MoS_2_ QDs could specifically fluorescently label the RCC cells and presented a distinct MRI signal enhancement in the tumor region of mice bearing RCC tumors [18]. Jingjing Li et al. further reported that the obtained poly (ethylene glycol) (PEG)-MnO nanoparticles displayed a high T_1_ relaxivity and a low r_2_/r_1_ ratio (12.942 s^−1^ mM^−1^ and 4.66) at 3.0 T, which was three times that of the clinical used contrast agent, Magnevist (Gd-DTPA), indicating the promising potential of PEG-MnO nanoparticles as T_1_ MRI contrast agent [79]. T_1_-T_2_ dual-modal MRI avoids the false-positive signals caused by a single imaging mode and provides more accurate and complementary information. However, interferences between the T_1_ and T_2_ CAs when they are in proximity would reduce their MRI relaxivity. Fe_3_O_4_@mSiO_2_/PDDA/BSA-Gd_2_O_3_ nano-complex was developed as a T_1_-T_2_ dual-mode CA (Fig. 3) [80]. mSiO_2_ nanoshell was employed to increase the distance between T_1_ CA, BSA-Gd_2_O_3_ nanoparticle and the T_2_ CA, Fe_3_O_4_ nanoparticle, because the interference of T_2_ CA on the contrast enhancement of paramagnetic T_1_ CAs might be weakened when their distance exceeds 12 nm. The obtained nanocomplex displayed high longitudinal (r_1_ = 11.47 mM s^−1^ Gd) and transverse (r_2_ = 195.1 mM s^−1^ Fe) relaxivities. To extend their utility as mMR imaging nanoprobes for the targeted detection of RCC tumor cells, the AS1411 aptamer was covalently bonded with the nano-complex, which could induce a kidney contrast-enhancement and was expelled via the bladder, demonstrating potential as a T_1_-T_2_ dual-mode CA.

In the late 1980s, Gd-DTPA as T_1_ MRI CA was approved by the FDA and European agencies for clinical practice [81]. Despite their widespread use, Gd-chelates designed to reduce Gd^3+^ toxicity demonstrated relatively low relaxivity, short blood circulation times, and non-specific bio-distribution [82]. It was discovered that nano-contrast agents possess higher water proton relaxivities, which was attributed to the densely populated metal ions in nanoparticles. Easy conjugation with targeting molecules and longer blood circulation time further favored their biological applications.

#### Nuclear medicine

Retrospective studies reveal that even small tumors have a potential for early lymphatic or distant metastatic spread. Lymphoscintigraphy is more sensitive than lymphangiography because its imaging agent is more consistent with physiological processes within the human body. Compared to lymphangiography, lymphoscintigraphy is more in line with the way the human body works and does not destroy the lymphatic channels directly. After injection, the nanocolloid is small enough to rapidly enter into lymphatic channels but are large enough to be trapped in lymph nodes. Subsequently, some studies performed that sentinel lymph node biopsy (SLNB) after intratumoral injection of ^99m^Tc-nanocolloid and imaging with scintigraphy and single photon emission computed tomography (SPECT)/CT in renal tumors is feasible [83, 84]. As has often been found for other tumor, the lymphatic drainage may not follow the known pattern. Thus, lymphatic mapping with planar lymphoscintigraphy is of great necessity. These could preoperatively identify lymph nodes draining directly from the primary tumor, especially outside the area of routine dissection [84, 85]. However, the percentage of non-visualization limited the use of SLNB for research and clinical purposes in renal cancer [86]. Further studies are needed to improve visualization and standardize the procedure of SLNB in renal tumors.

All in all, sequential lymphoscintigraphy using nanocolloid and sentinel node biopsy in RCC may enhance early detection of lymph node metastases without the associated morbidity of extensive lymph node dissection.

## Treatment of renal cell carcinoma using nanomedicine

We present the mechanisms of newly designed nanomedicine for treating RCC, mainly including image-guided surgery, targeted therapy, chemotherapy, radiotherapy, gene therapy, immunotherapy, and their synergetic therapy (Table 2). Combination therapy is the most actively pursued approach of present-day antitumor therapy. Current research has shown that nanomedicine has significant advantages over traditional techniques, such as selective medication delivery to cancerous cells and the avoidance of potential toxicity to normal cells. Therefore, efforts shall be made to translate these research insights into clinical practices for RCC.

**Table 2:** Overview of nanomedicine designed for treatment in RCC

Treatment strategies	Therapeutic agent	Applied nanoparticles	Experimental model	Key findings	References
Image-guided surgery	Activated excretion-retarded tumor imaging (AERTI) strategy	A near-infrared peptide probe (self-assembles into nanofibers)	786-O cell line and tumor-bearing nude mice	Achieve an accurate identification of the tumor boundaries and detection of minimal tumors	[88, 89]
	Intratumoral injections of ^99m^Tc-nanocolloid	^99m^Tc-nanocolloid	Clinical trial	Locate and sample the sentinel nodes at surgery	[83–85]
Targeted drugs	Sorafenib-loaded poly acid and DPPC liposome nanoparticles	PLGA, DPPC liposome and HMC-coated DPPC liposomes	786-O cell line	Optimize drug delivery and tumor cellular kill thereby improving the quality of Sorafenib-regimented therapy	[92]
	Liposomes encapsulating a multi-receptor tyrosine kinase inhibitor	Liposomes	769-P, ACHN, A498 and Caki-1cell lines and xenograft model	Improve intratumoral concentration, enhance antitumor efficacy and reduce toxicities	[93]
	Focused ultrasound-triggered release of tyrosine kinase inhibitor from thermosensitive liposomes	Thermosensitive liposomes	786-O cell line	Enhance drug delivery and cancer treatment by this combination	[94]
	Sorafenib combined with tumor hypoxia directed nanoparticles	Versatile tumor hypoxia directed nanoplatform	Human RCC A498 cells and its tumor model	Reverse drug-resistance and re-educe tumor-associated macrophages	[95]
	PEG-EGCG used as such a carrier forming Sunitinib-loaded micellar nanocomplex (SU-MNC)	Micellar nanocomplex	ACHN and A498 HRCC-xenograft models	The carrier-drug synergies with the high-performance carrier and tumor-targeted delivery	[96]
Chemotherapy	Liposomes encapsulating doxorubicin	Liposomes	OS-RC-2 cells and RCC xenograft mice model	Treat drug resistant RCC via the disruption of tumor endothelial cells	[100, 101]
	A novel tumor-targeted liposomal formulation loaded with everolimus and vinorelbine	Liposomal formulation	786-O, A498 cell lines and its xenograft model	Inhibit tumor growth and lung metastasis	[20]
	An oxygen nanocarrier combined with decitabine	Oxygen nanocarrier based on hemoglobin (H-NPs)	786-O, 769-P, Caki-1-1, ACHN, RCC4 cell lines, nu mice and human renal normal tumor samples	Alleviate resistance to oxaliplatin and decitabine in RCC cells under hypoxia	[102]
	Recognition-reaction-aggregation cascaded strategy and doxorubicin	Self-assembled superstructure (nanoparticles) on the cancer cell membrane	SK-RC-52 cell line and its tumor-bearing mice	Significantly inhibits the tumor growth	[19]
Radiotherapy	X-ray radiation and black phosphorus quantum dots	Black phosphorus quantum dots	786-O, A498 cell lines and tumor-bearing mice	Enhance ionizing radiation-induced apoptotic cell death of RCC cells	[104]
Native medicine	Plitidepsin	Polymer nanoparticles	MRI-H-121 cell tumor bearing mice	Present lower liver and kidney accumulation; reduce the growth rate of tumors	[105]
	Chlorogenic acid (CGA)	Chitosan nanoparticles (nano-sized colloidal delivery vector)	786-O cell line	Enhance antioxidant properties, intracellular accumulation, and antiproliferative activity	[106]
	Lupeol (Lup)	Polycaprolactone/Gelatin nanofibers	ACHN cell line	Enhance cytotoxicity activity by effective diffusion and elution to the target achieved	[107]
	Curcuma wenyujin	Gold nanoparticles	A498 and SW-156 cell lines	Activate proapoptotic proteins, inhibit antiapoptotic, thereby induce apoptosis	[108]
	Oudemansiella raphanipies polysaccharide	Selenium nanoparticles	786-O cell line	Cellular damage induced by ROS imbalance and mitochondria-mediated pathways	[109]
	Silibinin	Magnetic-core-based nanopolymeric carriers	A-498 cell line	Act as a potential carrier for targeted transportation of actives in cancer therapy	[110]
Photothermal therapy	Local heat under NIR irradiation	TiO_2_@red phosphorus nanorods (TiO_2_@RP NRs)	OS-RC-2, 786-O cells and tumor-bearing mice	Kill RCC cells by producing local heat and ROS	[112]
	The phase transition temperature and endoplasmic reticulum stress	tLyP-1/PR-619/Fe_3_O_4_@PCM (tPF@PCM)	786-O cell line and mouse xenograft model	Exacerbate endoplasmic reticulum stress, induce apoptosis and the favorable synergistic antitumor efficacy	[113]
	Local hyperthermia; lonidamine	Mesoporous silica nanoparticles	786-O cell line and RCC tumor-bearing mice	Enhance antiproliferative and tumor suppressing abilities	[114]
	Hyperthermic temperature	Gold nanorods	Caki-2 cell line	Dual capabilities as photothermal agents and autofluorescence enhancer to track cell death	[115]
	Laser thermal ablation; sorafenib	Gold nanorod encapsulated albumin	786-O cell line and mouse xenograft model	Significant synergistic tumor necrosis greater than each individual arm alone	[116, 117]
Gene therapy	pVHL complexes	Polyethyleneimine-derived nanoparticles	OS-RC-2 cell line and mouse xenograft model	Have increased transfection efficiency and obviously lower toxicities	[121]
	VEGFR (fms-like tyrosine kinase-1: sFlt-1)	Neutral lipid envelope-type nanoparticle	OS-RC-2-bearing mice	Promising gene carrier for targeting tumors for curing RCC	[122]
	AIM2 gene	H1/pAIM2 nanoparticles	786‐O and OS-RC‐2 cell lines; xenograft model	Inhibit malignancies of renal cancer through enhancing the inflammasome pathway	[123]
	Simultaneously inject Sorafenib and PH1/pHGFK1	PH1/pHGFK1 nanoparticles	Ketr-3, 786-O, and ACHN cell lines and tumor-bearing nude mice	Enhance anti-tumor activities of sorafenib and reverse its drug resistance evolution	[124]
	LNP formulation of siRNAs targeting VEGF and kinesin spindle proteins (ALN-VSP)	Lipid nanoparticles	Clinical trial	Experienced 12–18 months of tumor stabilization	[125]
	siRNAs	Multifunctional envelope-type nanodevice (MEND)	OS-RC‐2 cell line; mouse xenograft model	Deliver siRNA to a target cell in tumor tissue through an improved siRNA bioavailability	[126–128]
	siVEGF	Nanogel complex	RCC cell lines; xenograft model	Result in efficient knockdown of VEGF	[129, 130]
	siLim1	Polydiacetylenic nanofibers (PDA-NFs)	786-O cell line and mouse xenograft model	Efficiently silence the oncogene Lim-1; an innovative system for delivery of siRNAs	[131]
	siRNA	Purified glycogen polycationic derivatives (PGPD)	Renal cancer cells	The delivery of nucleic acids	[132]
	miRNA-143	Polyion complex (PIC)-loaded miRNA-143#12	Caki-1 cell line and mouse xenograft model	Induce a marked growth inhibition by impairing K-RAS-signaling networks	[133]
Tumor vaccine	H1-pAIM2/pCAIX vaccine; H1-pHMGB1/pB7H3 vaccine	H1 nanoparticles	HEK293T cell line and mouse xenograft model	Enhance tumor-specific multi-functional CD8 + T-cell responses	[136, 138]
	CS-pL-Myc/pCAIX vaccine	Chitosan nanoparticles	HEK293T cell line and mouse xenograft model	Induce multi-functional CD8 + T cell responses and inhibit lung metastasis	[137]
	Short peptide particles	Liposomes	RENCA cells and mouse xenograft model	A viable therapeutic approach via multivalent particle immunization	[139]

### Nanomedicine for image-guided surgery

**Fig. 4:**
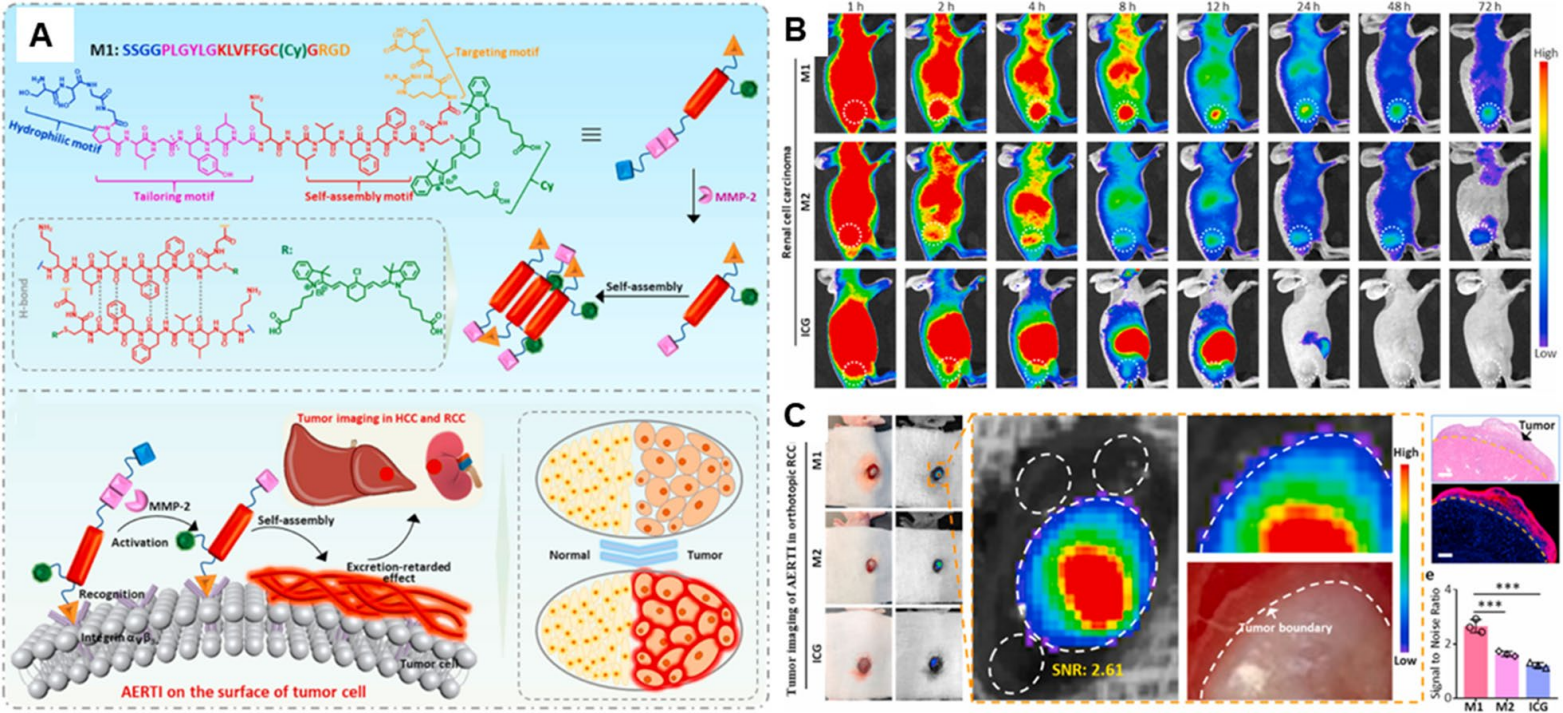
An activated excretion-retarded tumor imaging (AERTI) strategy was reported. In vivo verification of the tumor-selectively accumulation, retention and the SNR in tumor imaging of AERTI strategy. **A** Schematic illustration of the AERTI strategy. The AERTI strategy consisted of RGD, PLGYLG, KLVFFGC, Cy and SSGG. Upon MMP-2 specific enzymatic cleavage, the remaining molecule was triggered to form nanofibril conformation of antiparallel β-sheet. **B** Representative fluorescence images of 786-O xenograft mice after intravenous injection of M1, M2 and ICG. **C** Representative fluorescence images of RCC after intravenous administration in the orthotopic RCC xenograft mice at 24 h post injection. Calculate and statistical analyses SNR ratio. The tumor boundary identified by naked eyes, fluorescence imaging, H&E and fluorescence microscopy imaging results. Figure adapted from Da-Yong Hou et al. [89]

Surgical resection is still the foremost treatment for RCC patients. Maximal resection of the tumor is crucial for achieving the long-term disease control in clinic, which efficiently limits the recurrence and progression of tumor [7]. Therefore, it is of great significance to acquire adequate visualization of tumor boundaries in the medical surgery [87]. Intraoperative fluorescence-based tumor imaging could perform the oncological safe tumor resection with the advantage of differentiating tumor from normal tissues. Hongwei An et al. reported a near-infrared (NIR) peptide probe with a signal-to-noise ratio (SNR) of 2.5 [88]. The NIR peptide probe first recognized α_v_β_3_ integrin overexpressed in RCC cells, then was cleaved by MMP-2/9, which up-is regulated in the tumor microenvironment. The probe residue spontaneously self-assembled into a nanofibrous superstructure. Owing to this dual specificity of targeting and cleavage, high-performance identification of human RCC was achieved. This NIR peptide probe based on the TER strategy enabled precisely identifying tiny lesions (< 1 mm) that are eye-invisible in a standard bright field, then achieved complete tumor removal and significantly reduced postoperative recurrence compared with the traditional surgery group. In the subsequent work, they further demonstrated that the activated excretion-retarded tumor imaging (AERTI) was successfully accumulated at the tumor sites in the RCC xenograft models with a tumor retention time up to 72 h (Fig. 4) [89]. Besides, the lymphatic drainage may not follow the known pattern. As has been previously noted, sentinel node identification using intraoperative lymphoscintigraphy to locate and sample the sentinel node is feasible [83–85].

For the side effects of nano-strategy: Aggregation, polymerization, or assembly of biomaterials, unlike low-molecular weight drugs, can cause unexpected and unpredictable immunogenicity, which can lead to serious and life-threatening adverse effects. Fortunately, compared with antibodies, the benefit of peptides is their low immunogenicity. Nevertheless, before clinical translational application of peptide-based biomaterials, it is critical to elucidate the major risk factors that could lead to immunogenicity.

### Nanomedicine for targeted drugs

Targeted therapy is a popular modality of pharmacotherapy for cancer in recent years. As the name suggests, targeted drugs refer to the design of a corresponding therapeutic drug at the cellular and molecular level for a well-defined carcinogenic site (the site can be a protein molecule or a gene fragment in the tumor cell) [90]. Anti-angiogenic therapies are effective in metastatic RCC, but resistance and toxic side effects are inevitable [91]. The integration of nanoparticles in the treatment of RCC presents useful opportunities to overcome limitations of traditional targeted therapy. James Liu et al. developed and compared several Sorafenib-loaded biocompatible nanoparticle models [92]. Poly (lactic-co-glycolic) acid (PLGA), liposome, and hydrophobically modified chitosan (HMC)-coated liposomes could encapsulate Sorafenib with reproducibility. At maximum dosage and time (15 μM and 96 h), Sorafenib-loaded PLGA and HMC-coated liposomes killed 88.3 ± 1.8% and 98 ± 1.1% of all tumor cells, significant values compared with Sorafenib 81.8 ± 1.7%. PLGA particle due to uptake, possibly through a non-specific diffusion of nanospheres into cell membranes, may continuously release drug inside the cell through degradation thereby avoiding potential extracellular interactions and increasing availability of drug at a given dose. HMC modification improved cell uptake by increasing nanoparticle stability and, therefore, overall circulation time. Moreover, liposomes encapsulating multi-receptor tyrosine kinase inhibitor (XL184) induced sustained inhibition of tumor growth as compared to XL184 [93]. XL184 liposomes prevented opsonization, increased the circulation time resulting in an increased accumulation of drugs in the tumor and exhibited excellent stability in physiological buffer as well in plasma. However, some first-generation nanoparticles lacked capability for specificity and controlled drug release. Caleb Abshire et al. successfully created a TKI-loaded, thermosensitive liposomal nanoparticle capable of targeted drug release with focused ultrasound activation leading to increased tumor death. These findings might make the proposed combination of targeted chemotherapy, nanotechnology, and focused ultrasound a promising platform for enhanced drug delivery and cancer treatment [94]. According to analysis, the combined treatment led to the least viability, significantly lower than that observed from treatment with focused ultrasound or TKI/TSL at 96 h. To alleviate the drug resistance, Hashem O Alsaab et al. used Sorafenib in combination with tumor hypoxia directed nanoparticle loaded with a new class of apoptosis inducer, namely CAIX-C4.16 [95]. This versatile tumor hypoxia directed nanoplatform comprised of Vitamin-E-α-D-Tocopherol (TPGS) and styrene maleic anhydride (SMA) ligated with Acetazolamide (ATZ), namely CAIX-SMA-TPGS, which worked in synergy with existing drugs for reversing drug-resistance in RCC accompanied with re-education of tumor-associated macrophages. Due to its small molecular size and ease of chemical functionalization, CAIX-oligomer could be studied further for selective CAIX tumor targeting for diagnostic use in a clinical setting. The tumor spheroid uptake study clearly indicated that the CAIX-targeting oligomer has excellent tumor core penetration capacity, which is a significant signal of tumor stromal disruption leading to enhanced therapeutic response and immune modulation. If the nanocarrier has intrinsic therapeutic effects, the efficacy would be synergistically augmented. Poly (ethylene glycol)-conjugated epigallocatechin-3-O-gallate (PEG-EGCG) had been designed as such a carrier forming Sunitinib-loaded micellar nanocomplex (SU-MNC) [96]. EGCG, a major component of green tea, has been shown to possess anticancer effects. SU-MNC was shown to provide a significantly wider therapeutic window, demonstrating elevated anticancer efficacy and reduced systemic toxicity.

Nanoparticle-drug conjugates enhance drug delivery to tumors. Gradual payload release inside cancer cells augments antitumor activity while reducing toxicity. However, encapsulation of small molecule inhibitors in liposomes might be resulted in poor drug loading and burst release of the drugs from nanoparticles.

### Nanomedicine for chemotherapy and radiotherapy

RCC is notorious for its resistance to chemotherapy and radiation therapy in general, still common treatments for palliative management of metastatic RCC. Sensitization of chemo-drug response and overcoming radio-resistance become a new breakthrough point for RCC treatment.

Due to their long circulation time, PEGylated-liposomes (PEG-LPs) passively extravasate and accumulate in tumor tissues through leaky tumor vasculature by a universal mechanism called the enhanced permeability and retention (EPR) effect [97]. The intrinsic barriers generated by the extracellular matrix components in the tumor microenvironment control the penetration and distribution of PEG-LP in malignancies [98]. Due to the presence of PEG-LP in the deep tissues of hyperpermeable RCC tumors, doxorubicin (DOX)-loaded PEG-LP failed to induce anti-angiogenesis as well as an anti-tumor effect.

But it has been seen that ligand-based liposomes can be used to give chemotherapy by having peptides with the RGD or NGR motif that can target the neovasculatures [99]. Kazuhiro Takara et al. designed a dual-ligand LP encapsulating DOX [100]. The LPs were designed with a regulated diameter of roughly 300 nm and were modified with a particular ligand and a cell penetrating peptide (CPP) for targeting CD13-expressing neovasculature in RCC. As a specific ligand and CPP ligand, the LP membrane had an NGR motif peptide on top of PEG and tetra-arginine (R4) on the surface. The large size prevented extravasation of the dual-ligand LP, allowing it to associate with the target vasculature. The comparison revealed that tumor endothelial cells (TECs) were two orders of magnitude more susceptible to DOX than RCC cells, and tumor vascular disruption was efficiently induced. And the disruption of tumor vessels was efficiently induced. Unexpectedly, in the RCC tumor model, modification of the NGR motif had only a minimal influence on targetability and tumor growth. The findings of the further study indicated that large size PEG-LP, modified only with a single ligand RGD (RGD-PEG-LP) which interacts with Integrin α_v_β_3_, was efficient to both targeting and disrupting the tumor vasculature in RCC tissue [101]. Large-sized RGD-PEG-LP selectively targeted TECs by minimizing the EPR effect and significantly reduced tumor growth, which was exerted through its strong anti-angiogenic impact on tumor vasculature rather than having a direct effect on DOX-resistant RCC. Combination therapy is effective in alleviating the resistance mechanisms by targeting multiple signaling pathways but is usually more toxic than monotherapy. A tumor-targeted liposomal formulation was prepared using phospholipids, cholesterol, DSPE-(PEG)_2000_-OMe and a proprietary tumor-targeting-peptide-conjugated lipopeptide [20]. This study further demonstrated that the formulation, when loaded with everolimus and vinorelbine, was successful in inhibiting proliferation in vitro and tumor growth and lung metastasis in vivo.

Furthermore, human organic cation transporter 2 (OCT2) is the most abundant and important uptake transporter involved in the renal excretion of cationic drugs. Abnormal hypermethylation-mediated silencing of OCT2 results in oxaliplatin resistance in RCC. The epigenetic activation of OCT2 by decitabine (DAC) reversed the resistance in normoxic conditions. But the hypoxia-mediated repression of ENT1 led to the inability of DAC to upregulate the expression of OCT2 in hypoxic conditions. The hemoglobin-based oxygen nanocarriers H-NPs were constructed, with PLGA to load hemoglobin by hydrophobic interaction, and coated with a low molecular weight hydroxyethyl chitosan to target the kidney [102]. This study tested the role of H-NPs in DAC demethylation and detected a remarkable decrease in methylation frequency at the E-BOX motif, upregulated OCT2 expression, and increased oxaliplatin accumulation. H-NPs were shown to have a homogeneous dimension with small particle size, moderate aqueous phase stability, and efficient oxygen-carrying capacity. Moreover, currently supramolecular assemblies have been widely utilized to interact with biological membranes and help drugs enter cells, which is a way to enhance chemosensitivity [103]. A recognition-reaction-aggregation (RRA) cascaded strategy was utilized to in situ construct peptide-based superstructures on the RCC membrane [19]. Superstructure formed by RRA strategy could form a transient pore on the cell membrane, which enable the transport of compound. Based on above results, this study further demonstrated that RRA strategy could increase the influx of chemotherapy drug (DOX). Among that, RRA strategy showed a high S/N ratio and exhibited significant enhancement of accumulation on tumor site, suggesting its high specificity and stable retention capacity.

Palliative radiotherapy plays a valuable role in the management of metastatic RCC, especially for brain and painful bone metastasis. DNA is the principle cellular target for the biological effects of ionizing radiation. Yue Lang et al. found that black phosphorus quantum dots (BPQDs) inhibit DNA-PKcs activity and impair DNA-PKcs-mediated nonhomologous end joining DNA double-strand breaks repair, resulting in sustained DNA damage in response to ionizing radiation [104]. BPQDs enhances ionizing radiation -induced suppression of RCC xenografts growth in vivo.

These studies showed that combining nanoparticles with chemotherapy or radiotherapy is more beneficial than either monotreatment. RCC can no longer simply be recognized as chemo- or radio-resistant, and more studies are necessary for exploring their combination with other therapy strategies.

### Nanomedicine for native medicine

Bioactive molecules from native medicines or traditional folk medicines have been utilized as a complementary and alternative therapy for a variety of cancers. Conversely, some native medicine’s poor thermal and pH stability, poor solubility, and low cellular permeability have been a huge hindrance for it to exhibit its efficacy as a nutraceutical compound. Plitidepsin, an antineoplastic drug, is a cyclic depsipeptide originally isolated from the mediterranean tunicate Aplidium albicans. Hugo Oliveira et al. proposed two polymer-based nanoparticle systems, vesicular and micellar, as alternative approaches for plitidepsin delivery [105]. PEG-b-PBLG copolymer formed micellar structures, whereas PTMC-b-PGA formed vesicular structures. These copolymers allowed hydrophobic drugs to be solubilized and provided sustained release while improving biodistribution. Furthermore, the stealth character of the PEG moieties prevented interactions with cells and proteins, lengthening drug circulation time. Alternatively, a stable, non-toxic, monodispersed chitosan nanoparticles (CNP) synthesized via ionic gelation method at an optimum parameter (600 µL of 0.5 mg/mL of chitosan and 200 µL of 0.7 mg/mL of tripolyphosphate), denoted as CNP°, was used to encapsulate chlorogenic acid (CGA) [106]. The CNP could assist in enhancing its antioxidant properties, cellular accumulation, and increase chemopreventive efficacy at a lower concentration. Furthermore, Polycaprolactone/Gelatin (PCL-GEL) nanofibers were used as a drug delivery system. The PCL-GEL nanofibers containing Lupeol also showed high stability and anticancer activity [107]. The drug release profile confirmed the sustained release of about 80% achieved within 40 h. Furthermore, the biosynthesized C. wenyujin gold nanoparticles (CWAuNPs) [108] and oudemansiella raphanipies polysaccharide-decorated selenium nanoparticles (ORPS-SeNPs) [109] were potent anticancer agents which induce cell apoptosis. Their apoptotic pathway triggered in RCC cells was determined to be induced by reactive oxygen species (ROS) imbalance and mitochondria-mediated pathways and to eventually result in cellular oxidative stress damage. Besides, Anjali Takke et al. developed biocompatible magnetic-core-based nanopolymeric carriers of poly (D, l-lactide-co-glycolic) acid (PLGA) encapsulated silibinin (SLB) for the sustained release action [110]. SLB increased with the use of iron oxide nanoparticles through endocytic internalization into the cells. In such a delivery system, the synergy of magnetic fields and anticancer magneto-sensitive nanoparticle led to increase in anti-tumor activity.

Collectively, these nanoparticles can protect bioactive molecules from phagocytosis, deliver therapeutic drugs to targeted sites, provide an alternative route for insoluble drugs to permeate cells, improve drug bioavailability and therapeutic efficacy in living systems, upturn the EPR effect, participate in sustained drug release rates, and preserve drug pharmacodynamics and in vivo stability.

### Nanomedicine for photothermal therapy

**Fig. 5:**
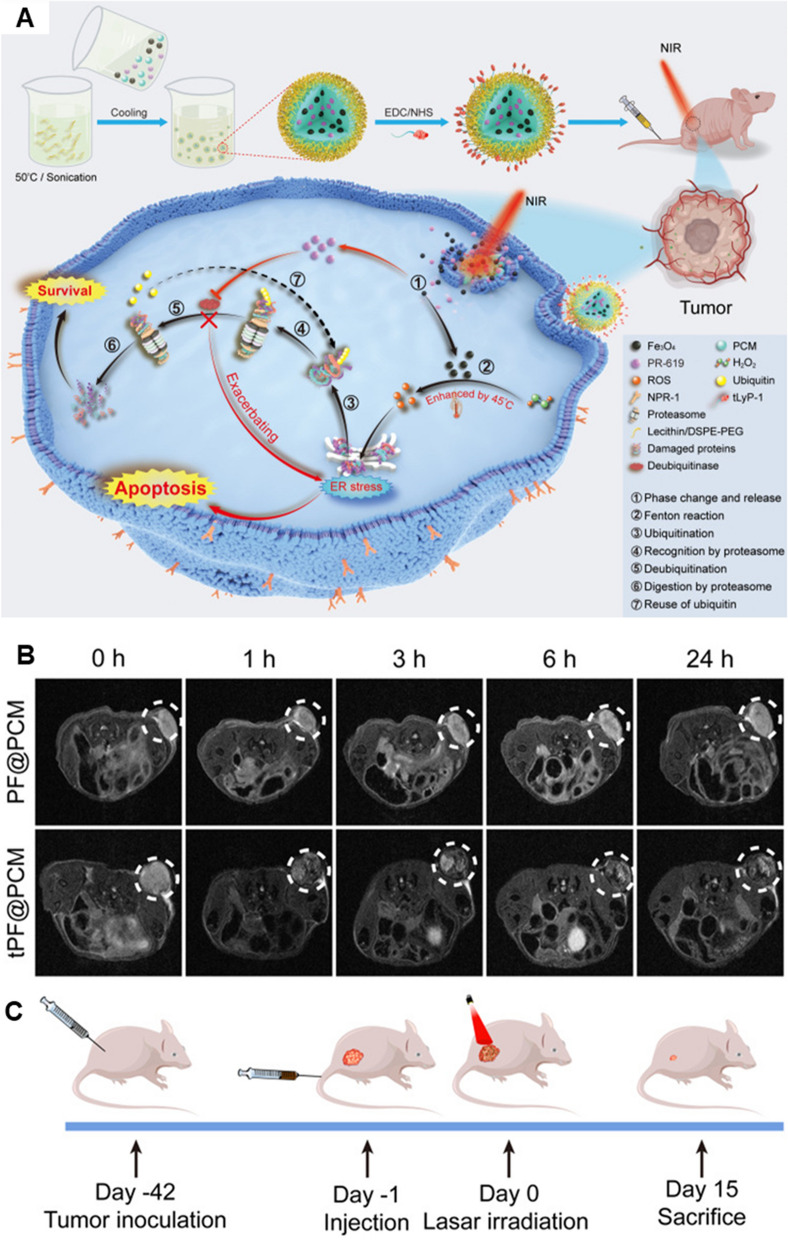
An efficient photothermal-augmented tumor therapeutic approach were developed, by regulating redox homeostasis concurrently with the activity of deubiquitinases, to convert the pro-survival unfolded protein response into the pro-apoptotic one. **A** Schematic illustration for the synthesis of tPF@PCM and the synergistic mechanism for cancer therapy. **B** T_2_-weighted MR images of tumor-bearing mice at 0, 1, 3, 6, and 24 h post-injection. **C** Schematic of the treatment regimen for 786-O tumor-bearing mice. Figure adapted from Biao Cai et al. [113]

Photothermal treatment (PPT), which kills cancer cells using nanomaterial-based phototoxicities, has gained a lot of attention in recent years. PTT uses the photothermal effect of photothermal transduction agents, which can turn light energy into heat to raise the temperature of the environment and kill cancer cells [111]. The core–shell structured TiO_2_ @red phosphorus nanorods (TiO_2_@RP NRs) as a photosensitizer were synthesized to drive PTT for RCC [112]. The optimized TiO_2_ @RP NRs could respond to NIR and produce local heat under irradiation. After NIR irradiation, TiO_2_@RP NRs efficiently killed RCC cells by producing local heat and ROS and cause low injury to normal kidney cells. Also, Biao Cai and his colleagues developed an efficient approach, by regulating redox homeostasis concurrently with the activity of deubiquitinases (DUBs), to convert the pro-survival unfolded protein response (UPR) into the pro-apoptotic one (Fig. 5) [113]. A nanocatalytic system, tLyP-1/PR-619/Fe_3_O_4_@PCM (tPF@PCM) co-loaded with Fe_3_O_4_ nanoparticles and the pan-DUB inhibitor PR-619, was synthesized by taking advantage of a melting point-controlled thermal responsive phase-change material (PCM). Fe_3_O_4_ nanoparticles were shown to be robust ROS inducers at the enhanced catalytic temperature after being released from tPF@PCM at 45 °C by laser irradiation, which enhanced the number of damaged proteins in the endoplasmic reticulum (ER) lumen and initiated ER stress. The increased input and simultaneously reduced output of ER stress were caught in a vicious circle, leading to prolonged activation of UPR, and ultimately causing apoptosis. Furthermore, the thermosensitive mitochondrial metabolism-interfering anticancer drug lonidamine was combined with the polydopamine (PDA) to treat RCC. Lonidamine and PDA were loaded in stellate mesoporous silica nanoparticles (MSNs) with a large surface area and cloaked with RCC membranes (MLP@M) [114]. When stimulated by NIR laser, PDA could generate a high temperature and destroy the cancer cell membrane, leading to lonidamine release and tumor-specific lonidamine accumulation.

Plasmonic PPT is emerging as a viable alternative to traditional laser therapy techniques due to its advantages in exploiting the unique properties of metal nanoparticles such as high photostability, reduced photobleaching, and increased absorption cross-sections when compared to traditional photothermal transduction agents. The potential of exploiting the dual capabilities of gold nanorods as photothermal agents and autofluorescence enhancer to track cell death [115]. As mentioned earlier, some studies have successfully encapsulated tyrosine kinase inhibitors (TKIs) in various nanoparticle carriers and demonstrated improved cellular kill [92]. Gold nanorods (AuNRs) have also been shown to function as drug-delivery vehicles, as they mediate drug release following NIR irradiation. Meanwhile, this research group has shown that combining TKI-loaded nanoparticles with AuNRs with laser activation can exhibit synergistic cell killing of RCC [116, 117].

PTT has the following advantages over other therapies: The use of external laser irradiation with adjustable dosage enables for accurate tumor targeting while minimizing damage to surrounding healthy tissues. It is well worth mentioning that some studies found that the hydrogel guaranteed the slow-release of the photosensitizer [118]. Therefore, the gel system avoided the side effects of the photosensitizer to healthy tissues. Moreover, Yuancun Cheng et al. designed that polypyrrole nanoparticles not only featured admirable photothermal conversion but also exhibited obvious photoacoustic imaging capability, which enabled imaging-guided enhanced tumor ablation [119]. These findings have meaningful clinical implications for giving new ideas for further investigation.

### Nanomedicine for gene therapy

The field of gene therapy has experienced an insurgence of attention for its widespread ability to regulate gene expression by targeting genomic DNA, messenger RNA (mRNA), microRNA (miRNA), and short-interfering RNA (siRNA) for treating malignant and non-malignant disorders. Numerous nucleic acid analogs have been developed to target coding or non-coding sequences of the human genome for gene regulation [120]. Theoretically, gene therapy is a simple therapeutic procedure that relies on either replacing a distorted gene with a healthy one or completing a missing gene to express the required protein.

#### DNA

Polyethyleneimine (PEI), which can form nanocomplexes with negatively charged DNA by electrostatic interaction, has been widely investigated as a gene delivery system. To improve their application value in gene delivery, Zhizhong Xu et al. demonstrated that three modified PEI-derived biomaterials had an increased transfection efficiency and a lower cytotoxicity compared with its precursor PEI [121]. It is worth mentioning that the mean tumor volume was obviously decreased 30% by using folic acid-PCFC-isophorone diidocyanate-PEI (FA-PEAs) to transfer VHL plasmids to treat RCC. Besides, a neutral lipid envelope-type nanoparticle composed of a pH-activated and vitamin E-scaffold lipid-like material was reported as a platform for a gene carrier targeting RCC [122]. When the particle's surface was modified for PEG, blood circulation stability improved, allowing tumor-selective gene expression to be achieved. When the hydrophobic scaffold of the ssPalm was replaced from the conventionally used myristic acid (ssPalmM) to -tocopherol succinate, there was a slight increase in gene expression in the tumor (ssPalmE). When the completely CpG-free pDNA encoding the solute form of vascular endothelial growth factor receptor (VEGFR) was used, tumor growth was significantly suppressed, especially when it was delivered by the liposomal nanoparticle (LNP) formed with ssPalmE. It was also found that absent in melanoma 2 (AIM2) expression was significantly decreased in RCC patient specimens and renal carcinoma cell lines. The nanoparticle consisting of a folate grafted H1 nanoparticle‐mediated AIM2 gene (H1/pAIM2) was formed [123]. H1/pAIM2 delivery in RCC cells could remarkably increase AIM2 expression, reducing cell proliferation, migration, and invasion while increasing cell apoptosis. As a gene delivery vector, H1-formed nanoparticle exhibits effective gene delivery and low cytotoxicity. Also, a novel cationic polymer-PH1 was combined with a potent anti-angiogenic factor (HGFK1), and its combination therapy with sorafenib was further investigated [124]. Intravenous injection of PH1/pHGFK1 nanoparticles significantly inhibited tumor growth and prolonged the survival time of tumor-bearing mice. Recombinant HGFK1 accelerated sorafenib-induced apoptosis and cell cycle arrest. And HGFK1 could also decrease sorafenib-induced autophagy and stemness via blockading the NF-κB signaling pathway in RCC. These results provided rational basis for clinical application of combination therapy in RCC patients.

The main purpose of all of these pharmaceutical developments is to make sure that the treatment has the best medical effects and the fewest side effects possible, in which case DNA is the drug to be administered. Efficient nano-delivery systems could be capable of protecting and delivering the transgene to target cells in which it can successfully express itself.

#### RNA

Gene silencing is a broad term for the epigenetic process of regulating genes. It is often used to describe the "shutdown" of a gene by a process other than changing its DNA.

The common mechanism of post-transcriptional gene silencing is RNA interference (RNAi). Therapy using RNAi is one of the most successful new frontiers of gene therapy, as it can act on almost all genes, influencing their behavior. Josep Tabernero et al. initiated a first-in-humans trial of an RNAi therapeutic targeting VEGF and KSP [125]. Three patients with RCC or pancreatic neuroendocrine tumor experienced 12–18 months of tumor stabilization. Besides, a multifunctional envelope-type nanodevice (MEND) with a PEG-peptide-DOPE ternary conjugate and a short GALA was designed to carry siRNA to tumor tissues in the body [126]. The administration of the MEND showed about a 50% reduction in the target gene mRNA and protein. They further reported on a combination therapy involving the use of siRNA-mediated specific gene knockdown and cytotoxic drug DOX, resulted in a measurable delay in RCC growth [127]. They also reported on the development of a system that permits the delivery of siRNA to TECs by combining the YSK-MEND and a ligand that is specific to TECs. RGD-MEND induced a significant RNAi-mediated gene reduction in TEC but not in endothelial cells of other organs [128]. VEGF is considered to be involved in the process of new vasculature formation. The VEGF-specific siRNA (siVEGF)/nanogel complex was engulfed by RCC cells through the endocytotic pathway, resulting in efficient knockdown of VEGF [129, 130]. Additionally, PDA nanofibers (PDA-Nfs) obtained by photopolymerization of surfactant 1 were optimized for intracellular delivery of siRNAs [131]. PDA-Nfs/siRNA complexes efficiently silenced the oncogene Lim-1 in RCC cells. Also, new cationic derivatives of the biocompatible polymer Purified Glycogen (PG) have been successfully prepared [132]. PGPDs-siRNA complexes show that they maintain their spherical and dendrimeric structure, that they can cross cellular barriers and act directly on the nucleus of the different cell lines. The percentage of nucleic acid that is released into the cell should be further increased. miRNAs are small noncoding RNAs that can bind to the 3′ UTR of the targeted mRNA, thus inhibiting translation or promoting RNA degradation. The treatment by RNAi using synthetic miRNA-143 loaded in the polyion complex nanocarrier exhibited a great anti-cancer effect when administered systemically [133]. This synthetic miRNA-143#12 induced a marked growth inhibition by impairing K-RAS-signaling networks.

RNAi conveys an alternative genetic approach for cancer patients, especially when conventional medications fail. The success of therapy is highly dependent on gene delivery nano-vectors that guarantee nucleic acids are efficiently internalized into target cells, which involves the inhibition of expression of specific mRNA that signals for uncontrolled cell growth and proliferation.

### Nanomedicine for tumor vaccine

Tumor vaccines are a potential treatment strategy for cancer immunotherapy by inducing antigen-specific T-cell immune responses [134]. DNA vaccination remains an approach to stimulate CD8 + T-cell responses. However, inefficient delivery of DNA remains a major issue. In an earlier study, PEI was used as a DNA vector carrier to improve the transfection efficiency of the DNA vaccine and stimulate humoral and cellular immunity against the renal carcinoma-associated antigen G250 [135]. A protein vaccine was included in the immunization strategy in order to produce a prime-boost effect. Also, H1 as a vehicle of nonviral gene could effectively condense DNA vaccine to form stable functionalized nanoparticles for vaccine delivery. Chai et al. designed a H1 nanoparticle-mediated DNA vaccine containing an adjuvant of AIM2 and a tumor-specific antigen of CAIX (H1-pAIM2/pCAIX) [136]. They also developed a chitosan nanoparticle-mediated DNA vaccine containing an activated factor L-Myc and a tumor-specific antigen CAIX [137]. Furthermore, the adjuvant HMGB1 and H1 nanoparticle delivery system would be further used [138]. These studies showed that the intramuscular administration of the vaccines could significantly inhibit tumor growth by enhanced tumor-specific CTL responses and multi-functional CD8 + T-cell responses. Recently, conversion of 20 short major histocompatibility class I (MHC-I) restricted neoepitope candidates into immunogenic nanoparticles could result in antitumor responses with multivalent vaccination [139].

A successful and effective vaccine relies mainly on antigen adjuvant and delivery system. In brief, stable functionalized nanoparticles-based delivery system may optimize the therapeutic effect of DNA vaccine for tumor treatment, which could stimulate the body to produce stable and high levels of humoral and cellular immune responses. However, the specificity of the immune response induced by DNA vaccine was needed to be verified by further histology analysis of tumor and other non-target organs.

## Conclusion and further perspective

In this review, we have highlighted the most advanced progress on the application of nanomedicine in RCC management, from imaging of kidney lesion to treatment of kidney cancer. Although the late diagnosis and poor prognosis of RCC lead to limited treatment options and an extremely low survival rate, nanomedicine can overcome these challenges and apply relevant research findings for RCC diagnosis and treatment. We could control the interaction of nanomaterials with target cells, by manipulating the sizes, shapes, surface charges, and composition of nanoparticles. Many studies and clinical trials have yielded many successful nanomedicines for systematic management of kidney cancers for diagnosis and therapy. AgNPET with mass spectrometry for analysis and imaging abilities showed differentiation between normal and cancerous renal tissue, which is thus highly promising to move from the bench to the bedside. Targeted nanobubbles can penetrate the tumor vasculature and achieve ultrasound molecular imaging of tumor parenchymal cells. Various metallic nanomaterials are used as contrast agents for MRI providing a higher probability of detecting early kidney cancer. An AERTI strategy with an extended tumor retention time and enhanced SNR enables precisely identifying eye-invisible tiny lesions, which contributed to complete tumor removal. Many other biocompatible nanosystems that target cancer have also demonstrated efficacy in therapy. Nanomedicine has already revolutionized the way we discover and administer drugs in biological systems. Furthermore, it is worth mentioning that development of agents that enable simultaneous real-time diagnostic (imaging) and therapeutic (drug delivery), also known as theranostic agents, has gained increasing attention. The use of nanotheranostics causes a drastic increase in therapeutic efficiency by allowing the researcher or physician to monitor the accumulation of drugs at the active site and the timing to trigger drug release at the target site [140–142]. These accumulated experiences provide a strong foundation for the future development of innovative nanomedicines for cancer imaging and treatment.

Nevertheless, we should notice most developments and new findings in this area have not been validated in clinical trials yet. There are several challenges to overcome for the successful development of nanosystems to achieve cancer management in clinical settings. One of the main challenges concerns the in vivo behavior of nanoparticles, which is likely to differ greatly from their in vitro behavior. We need to focus on cellular interactions, tissue transport, diffusion, and biocompatibility. Using many animal models to provide sufficient evidence of efficacy and safety is neither simple nor inexpensive. The complexity and heterogeneity of tumors is another challenge. Different types of cancer may have different gene expression profiles, molecular patterns, and drug resistance, which could make it harder for nanoparticles to get into the cancer cells and make them less effective. Last but not least, if nanomedicines are scalable to mass production to aid clinical cancer management, high standards will be required for nanomaterial synthesis reproducibility and the impact on systematic preclinical toxicity evaluation and human trials.

Overall, we think that the increasing rate of cancer-related deaths is the driving force behind the expected global nanomedicine market size increase in coming years. Cooperation between the clinical, medical and the scientific fields is urgently required to further expedite the clinical translation of nanomedicines for efficient imaging and treatment for RCC patients.

## Data Availability

Not applicable, please refer to the original references.

## Abbreviations

RCC: Renal cell carcinoma
CT: Computed tomography
MRI: Magnetic resonance imaging
US: Ultrasonography
QD: Quantum dot
MS: Mass spectrometry
AuNPET: Gold nanoparticle enhanced target
SALDI: Surface assisted-laser desorption/ionization
^1^H NMR: High resolution proton nuclear magnetic resonance spectroscopy
MALDI: Matrix-assisted laser desorption/ionization
LARESI: Laser ablation-remote-electrospray ionization
IHC: Immunohistochemistry
IF: Immunofluorescence
miRNAs: MicroRNAs
PCR: Polymerase chain reaction
γ-PNA: γ-Peptide nucleic acid
EVs: Extracellular vesicles
TEM: Transmission electron microscopy
NTA: Nanoparticle tracking analysis
CTCs: Circulating tumor cells
SERS: Surface-enhanced Raman spectroscopy
USMI: Ultrasound molecular imaging
CAs: Contrast agents
LN: Lymph node
MNP: Lymphotropic magnetic nanoparticles
MFLs: Magnetic fluid-loaded liposomes
T_1_: Longitudinal relaxation time
T_2_: Transverse relaxation time
PEG: Poly (ethylene glycol)
SPIO: Superparamagnetic iron oxide
mAb: Monoclonal antibody
SLNB: Sentinel lymph node biopsy
SPECT: Single photon emission computed tomography
NIR: Near-infrared
SNR: Signal-to-noise ratio
AERTI: Activated excretion-retarded tumor imaging
PLGA: Poly (lactic-co-glycolic) acid
HMC: Hydrophobically modified chitosan
TPGS: Vitamin-E-α-D-Tocopherol
SMA: Styrene maleic anhydride
ATZ: Acetazolamide
PEG-EGCG: Poly (ethylene glycol)-conjugated epigallocatechin-3-O-gallate
SU-MNC: Sunitinib-loaded micellar nanocomplex
PEG-LPs: PEGylated-liposomes
DOX: Doxorubicin
EPR: Enhanced permeability and retention
CPP: Cell penetrating peptide
TECs: Tumor endothelial cells
OCT2: Organic cation transporter 2
RRA: Recognition-reaction-aggregation
IR: Ionizing radiation
BPQDs: Black phosphorus quantum dots
CNP: Chitosan nanoparticles
PCL-GEL: Polycaprolactone/gelatin
PLGA: Poly (D, l-lactide-co-glycolic) acid
ROS: Reactive oxygen species
PPT: Photothermal treatment
TiO_2_@RP NRs: TiO_2_ @red phosphorus nanorods
DUBs: Deubiquitinases
UPR: Unfolded protein response
PCM: Phase-change material
ER: Endoplasmic reticulum
PDA: Polydopamine
MSNs: Mesoporous silica nanoparticles
TKIs: Tyrosine kinase inhibitors
mRNA: Messenger RNA
siRNA: Short-interfering RNA
RNAi: RNA interference
PEI: Polyethyleneimine
FA-PEAs: Folic acid-PCFC-isophorone diidocyanate-PEI
VEGFR: Vascular endothelial growth factor receptor
MEND: Multifunctional envelope-type nanodevice
MHC-I: Major histocompatibility class I
